# Effects of different personal protective equipment on the cardiovascular system in a 6-minute walk test

**DOI:** 10.1016/j.clinsp.2026.100994

**Published:** 2026-05-06

**Authors:** Padmavathy Kathamuthu Masilamani, Noorzaid Bin Muhammad, Rohith Sharan Sankaran, Resni Mona, Pradeep Palanisamy, Theingi Maung Maung

**Affiliations:** a Cluster for Integrative Physiology and Molecular Medicine (CIPMM), Faculty of Medicine, Universiti Kuala Lumpur, Royal College of Medicine Perak, Malaysia; b Provas Medical Centre and Research Institute, India; c Centre for Medical Education, University of Dundee, United Kingdom; d Faculty of Medicine, Universiti Kuala Lumpur, Royal College of Medicine Perak, Malaysia

**Keywords:** 6-Minute Walk, PPE, Facemask, SpO_2_, Heart Rate, Blood pressure

## Abstract

**Introduction:**

During the Coronavirus Disease (COVID-19) pandemic, public and healthcare workers were recommended to use Personal Protective Equipment (PPE) to prevent the spread of infection. With the increased use of different types of respiratory protection, including facemasks and PPE, cardiovascular parameters are expected to vary within physiological levels.

**Objective:**

This study aimed to evaluate the variations in cardiovascular parameters on wearing facemasks or full-body PPE while performing the 6-Minute Walk Test (6-MWT), a standardized physical exercise.

**Methods:**

This quasi-experimental study involved 99 healthy young adults aged 18–24 years with a normal mean BMI. The participants were randomly selected into a control and two experimental groups (wearing a facemask, or full-body PPE). Measurements of Heart Rate (HR), Blood Pressure (BP), and Oxygen Saturation (SpO_2_%) were performed before and after 6-MWT. The data were analyzed using paired *t*-tests for comparison of parameters and repeated measures ANOVA for a Group × Time (Pretest/Posttest) interaction between the groups, where p-value < 0.05 was considered statistically significant.

**Results:**

Based on paired *t*-tests, heart rate and blood pressure showed a significant difference between pretest and posttest in each group. Mixed-model ANOVA revealed no significant Group × Time interactions for any cardiovascular parameter, indicating that the physiological response to the 6-MWT was similar regardless of PPE worn. A significant difference in 6-minute walk distance was observed between the facemask group and full-body PPE group.

**Conclusion:**

In this cohort, no significant effects of Group × Time interaction were observed for SpO_2_%, HR, and BP on wearing different PPE and performing 6-MWT, a submaximal physical activity.

## Highlights

• Wearing full-body personal protective equipment does not reduce oxygen saturation.
• Effects of full-body personal protective equipment are similar to wearing a facemask.
• Human body acclimates to personal protective equipment within physiological limits.
• The benefits of wearing respiratory protection outweigh any perceived risk.

## Graphical abstract

**Figure:**
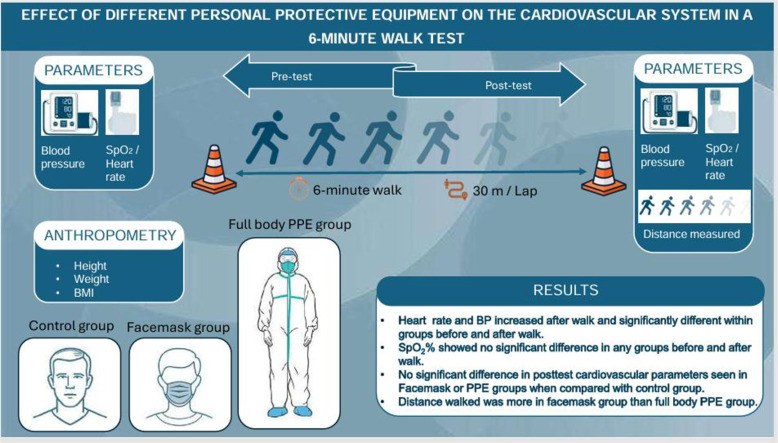

## Introduction

The Coronavirus Disease (COVID-19) pandemic had an impact on human life, prompting public and healthcare workers to wear protective respirators, including facemasks and Personal Protective Equipment (PPE), to reduce potential exposure risk of infection. Physical inconvenience and physiological consequences were noted to occur when wearing face masks, face shields, or full-body PPE for an extended period.^1^ Notably, the cardiopulmonary system adapts to acute physical exertion, which necessitates the calculation of the extent of Oxygen Saturation (SpO_2_), heart rate, and blood pressure changes due to usage of PPE.^2^

Evidence showed that the impact of using N95 Filtering Face-piece Respirator (FFR) among healthcare workers resulted in increased CO_2_ levels, shortening of breath, headache, dizziness, tachycardia, which resulted in a higher perception of physical exertion, thus impeding communication, affecting their performance and efficiency.^3^ Due to the longer usage of FFR, the effects of hypoxic and hypercapnic symptoms, including local symptoms of excessive sweating, drying, itching, and burning sensation of the nose, redness, and swelling of the face etc. were reported.^4^^,^^5^ Earlier studies had studied the changes in cardiopulmonary parameters such as oxygen saturation, pulse rate, and end-tidal CO_2_ during a physical activity of brisk walking for 10 min while wearing a cloth or surgical facemask.^6^ Later studies have found that cardiopulmonary exercise capacity was reduced more by wearing N95 masks than surgical masks on an incremental exertion test on a semi-recumbent ergometer.^7^ An impairment of cognitive function was also reported along with cardiopulmonary effects and metabolic shift due to rebreathing CO_2_ and hypoxaemia.^8^ An increase in heart rate was noted when wearing respirators at variable durations, a significant decrease in SpO_2_%, but no significant respiratory rate changes among surgeons wearing N95 masks during long procedures were found between baseline, at 3-hours, and 6-hours post-donning.^9^ PPE-associated discomfort, such as headaches or pre-existing headache disorders, was observed among healthcare workers.^10^ Another study, which was carried out among healthcare workers who wore an N95 facemask, focused on cardiopulmonary changes after 1-hour of treadmill exercise, found changes similar to existing evidence.^11^ One approach for evaluating the outcome of physical activity with a facemask or full-body PPE is the 6-Minute Walk Test (6-MWT) to assess the submaximal level of functional capacity. The 6-MWT is a great tool to assess the degree of functional impairment of the cardiopulmonary system while performing an activity. Practically, 6-MWT is used to evaluate functional capacity among normal and chronic cardiopulmonary patients; the exertion is observed to mimic the physical exertion in a natural working environment.^12^ Previous studies were conducted to evaluate the effects of 6-MWT on wearing facemasks found no significant difference in 6-Minute Walking Distance (6-MWD), heart rate, respiratory rate and SpO_2_% between those wearing a surgical mask or N95 mask, except for breathing difficulty.^13^ But significant increase in pulse rate, respiratory discomfort due to increased end tidal CO_2_ among healthy adults wearing a KN95 mask with a 3ply mask over it and wearing anti-fog goggles, but no significant changes in blood pressure and oxygen saturation on physical exertion of a brisk walk for 10 min.^14^ Further, the effects of full-body PPE on cardiovascular parameters among healthy adults with 6 MWT were not studied.

This study aimed to determine the effects of using a facemask (KN95) and a full-body PPE on cardiovascular parameters such as blood pressure, heart rate and oxygen saturation among young adults when performing 6 MWT, a validated physical activity method.

## Materials and methods

### Study design and sampling

This study employed a quasi-experimental design as proposed by Chang et al.^15^ Sample size was calculated to find the difference in the study population with a 95% Confidence Interval, taking α = 0.05, β = 0.2 (Power = 80%), for a paired difference comparison between the baseline and the posttest value of SpO_2_%, from a previously published study.^16^ Assuming an attrition rate of 20% between the pretest and post-test measurement, the total sample required for the study was calculated to be 33. The sampling frame was medical students of Universiti Kuala Lumpur (UniKL), Royal College of Medicine Perak, Malaysia. Participation was voluntary and the subjects were randomly selected into three groups, each with thirty-three participants: Two experimental groups (facemask group and PPE group) and one control group to compare post-intervention outcomes following a 6-MWT. As participants were not informed about their groups prior to the study. However, participants were not blinded after assigning in each group.

A pilot study was conducted with approximately 10% of the study sample to assess the risks and identify any potential confounding elements. Both pilot and main studies were conducted in the same environment, in the premises of Universiti Kuala Lumpur, Royal College of Medicine Perak, Malaysia.

### Materials

The facemask used in this study was unvalved 3M 9501+KN95 (China) AS/NZS 1716 P2 (Australia/New Zealand) with a white ear loop. Along with the unvalved 3M 9501+KN95 facemask, full-body PPE included a head cap, shoe cover, body drape (Executive standard GB190822009, Hean Pharmaceutical Supervision Production, License No 20200216), face shield, and latex-unsterilized hand gloves.

Measurements of height (cm) and weight (kg) were taken. Cardiovascular parameters such as heart rate (beats per minute), blood pressure (mmHg), and transcutaneous Oxygen Saturation (SpO_2_%) were measured before and after walking, along with the distance walked (meters) after the test. A calibrated stadiometer for height and weight; a fingertip pulse oximeter (Model:C101A2; iMDK, China) for SpO_2_% and heart rate; and a calibrated aneroid sphygmomanometer for blood pressure were used to measure the parameters.

### Inclusion and exclusion criteria

The inclusion criteria included participants of healthy adults aged above 18-years, who had received both doses of the COVID vaccine, had no symptoms or recent pulmonary infection within the last month, and resided in Ipoh, Perak State. Participants with underlying cardiovascular or pulmonary illness or symptoms of active COVID-19 were excluded.

### Data collection

After explaining the procedure of the 6-MWT, informed consent was obtained from all participants, and personal details such as age, gender, contact number, and address were collected. Self-answering data collection questionnaire included details regarding COVID-19 vaccination status and any medical illness/recent surgery. Pretest and posttest measurements of heart rate, SpO_2_%, and blood pressure were taken, with the intervention being a 6-MWT. As the measurements were implemented using standardized equipment with appropriate calibration, bias related to assessors was limited. Medical students who were not enrolled in the study were the assessors and were not informed about the participants’ group before the study. Outcome assessors and data analysts were not blinded due to the nature of the intervention. The average ambient temperature during data collection was 27±2°C and the humidity ranged between 85±2%.

The 6-MWT was performed according to the recommendations of the American Thoracic Society.^12^ The participants were required to walk as fast as possible for six minutes without running. During the walk, they were encouraged every minute to continue at the same pace and not to stop unless they felt any discomfort. The present study’s hypothesis was that the use of a KN95 facemask or a full-body PPE would have no significant difference on physiological cardiovascular parameters. The STROBE framework was used for complete and adequate reporting of this study.^17^

### Analysis

The data was analyzed using SPSS version 25.0. The normality of the data was assessed, mean and standard deviation. Pretest and posttest comparison of cardiovascular parameters were done using a paired *t*-tests. A mixed-design (or repeated measures) ANOVA was conducted with Group (Control, Facemask, PPE) as the between-subjects factor and Time (Pre-test, Post-test) as the within-subjects factor to test for Group × Time interaction effects. A p-value < 0.05 was considered statistically significant.

## Results

This study aimed to explore the variations in the cardiovascular parameters when wearing a KN95 facemask and a full-body PPE during controlled physical activity. Ninety-nine participants were randomly grouped into a control and an experimental group (a group wearing a KN95 facemask, and a group wearing full-body PPE), where each group had 33 participants. The normality of the data was assessed, where all parameters were normally distributed except oxygen saturation. The mean values of age, gender, and anthropometric parameters are presented in Table 1. As the groups were randomly selected from the medical students, the distribution of gender cannot be controlled, which is a limitation of this study.

**Table 1:** Mean values of age and anthropometric parameters among the groups.

Parameters	Mean±SD		
	Control group (*n* = 33)	Facemask group (*n* = 33)	PPE group (*n* = 33)
Age (years)	21.12 ± 1.93	21.52 ± 2.02	21.00 ± 1.37
Height (cm)	160.59 ± 0.46	160.49 ± 70.44	161.60 ± 7.30
Weight (Kg)	57.31 ± 13.02	61.11 ± 15.60	59.55 ± 15.97
BMI (Kg/m^2^)	22.37 ± 4.63	23.49 ± 5.07	22.55 ± 4.50
Gender			
Male (n)	7	8	11
Female (n)	26	25	22

The results obtained from paired *t*-tests for comparison of pre-test and post-test mean values of cardiovascular parameters among three groups are presented in Tables 2, Table 3, Table 4. All groups showed an increase in mean values of heart rate, systolic blood pressure and diastolic blood pressure after walking, showing significant differences (p < 0.05), while SpO_2_% decreased slightly, but did not show a significant difference between pretest and post-test values.

**Table 2:** The differences in cardiovascular parameters before and after a 6-Minute Walk Test (6-MWT) among control group (*n* = 33).

Cardiovascular parameters		Mean ± SD	95% CI of the difference		p-value
			Lower bound	Upper bound	
**Heart rate (BPM)**	Before walk	89.09 ± 12.69	−20.08	−10.23	0.000^a^
	After walk	104.24 ± 17.18			
**Systolic blood pressure (mmHg)**	Before walk	118.94 ± 9.33	−13.43	−6.87	0.000^a^
	After walk	129.09 ± 9.17			
**Diastolic blood pressure (mmHg)**	Before walk	74.85 ± 10.19	−10.96	−3.47	0.000^a^
	After walk	82.06 ± 9.57			
**SpO_2_ (%)**	Before walk	98.24 ± 0.61	−0.05	0.84	0.079
	After walk	97.85 ± 1.12			

a Significant difference at 0.001level.

**Table 3:** Differences in cardiovascular parameters before and after 6-Minute Walk Test (6-MWT) among facemask group (*n* = 33).

Cardiovascular parameters		Mean ± SD	95% CI of the difference		p-value
			Lower bound	Upper bound	
**Heart rate (BPM)**	Before walk	89.85 ± 12.69	−25.19	−15.42	0.000^a^
	After walk	110.15 ± 16.09			
**Systolic blood pressure (mmHg)**	Before walk	117.58 ± 11.34	−17.89	−7.45	0.000^a^
	After walk	130.24 ± 13.14			
**Diastolic blood pressure (mmHg)**	Before walk	75.97 ± 9.93	−10.93	−3.32	0.001^a^
	After walk	83.09 ± 7.93			
**SpO_2_ (%)**	Before walk	98.30 ± 0.85	−0.12	0.77	0.133
	After walk	97.97 ± 0.88			

a Significant difference at 0.001 level.

**Table 4:** Differences in cardiovascular parameters before and after a 6-Minute Walk Test (6-MWT) among full-body PPE group (*n* = 33).

Cardiovascular parameters		Mean ± SD	95% CI of the difference		p-value
			Lower bound	Upper bound	
**Heart rate (BPM)**	Before walk	85.70 ± 11.53	−28.05	−15.89	0.000^a^
	After walk	107.35±17.41			
**Systolic blood pressure (mmHg)**	Before walk	116.67±12.96	−17.40	−9.21	0.000^a^
	After walk	129.97±14.35			
**Diastolic blood pressure (mmHg)**	Before walk	76.09±11.77	−9.37	−0.81	0.021^b^
	After walk	81.18±13.27			
**SpO_2_ (%)**	Before walk	98.36±0.65	−0.11	0.53	0.182
	After walk	98.15±0.67			

a Significant difference at 0.001.

b Significant difference at 0.05.

A mixed-model (repeated measures) ANOVA for Group × Time interaction effects with Group (Control, Facemask, PPE) as the between-subjects factor and Time (Pre-test, Post-test) as the within-subjects factor is shown in Table 5. The results indicated that there is no significant difference between control and intervention groups (Facemask and PPE groups) related to Oxygen Saturation (SpO_2_%), blood pressure, and heart rate. Mauchly’s test of sphericity was violated (p > 0.05) for all cardiovascular parameters and degrees of freedom were corrected using Huynh-Feldt estimates of sphericity (ε = 1) since Greenhouse-Geisser epsilon was >0.75. However, Table 5 shows the critical finding that the Group × Time interaction for SpO_2_% is non-significant (p = 0.805), meaning the minor change within the facemask group was not different from the change in other groups.

**Table 5:** One-way repeated measures ANOVA test results for a Group × Time (Pre/Post) interaction among groups.

Cardiovascular parameters	Groups	Mean ± SD		F	p-value	ηp^2^	Wilks' Lambda
		Pre-test	Post-test				
Heart rate (BPM)	Control	89.09 ± 12.68	104.24 ± 17.12	Time (160.84)	0.000	0.626	0.374
	Facemask	89.85 ± 12.65	110.15 ± 16.09	Time*Group (1.849)	0.163	0.037	0.963
	PPE	85.70 ± 11.53	107.67 ± 17.41				
Systolic blood pressure (mmHg)	Control	118.94 ± 9.33	129.09 ± 9.17	Time (98.792)	0.000	0.507	0.493
	Facemask	117.58 ± 11.34	130.24 ± 13.14	Time*Group (0.631)	0.534	0.013	0.987
	PPE	116.67 ± 12.96	129.97 ± 14.35				
Diastolic blood pressure (mmHg)	Control	74.85 ± 10.19	82.06 ± 9.56	Time (33.465)	0.000	0.258	0.742
	Facemask	75.97 ± 9.93	83.09 ± 7.93	Time*Group (0.383)	0.874	0.008	0.992
	PPE	76.09 ± 11.77	81.18 ± 13.27				
SpO_2_ (%)	Control	98.24 ± 0.61	97.85 ± 1.12	Time (7.471)	0.007	0.072	0.928
	Facemask	98.3 ± 0.85	97.97 ± 0.88	Time*Group (0.218)	0.805	0.005	0.995
	PPE	98.36 ± 0.65	98.15 ± 0.67				

SD, Standard Deviation; *F*, Test Statistics; p-value, Probability; ηp^2^, Partial eta-squared.

The 6-Minute Walk Distance (6-MWD) among the groups (control group = 451.41±56.28 m; facemask group = 456.28±47.09 m; PPE group = 426.08±56.3 m) was analyzed by one-way repeated measures ANOVA and compared between groups. A significant difference in 6-MWD was found between the facemask group and the full-body PPE group (*F* = 3.043, p = 0.045, 95% CI 0.09–61.51). Post-hoc analysis revealed that participants wearing PPE walked 30 m less than the facemask group. Eta-squared showed a small effect size (η^2 =^ 0.060), indicating a weak difference between groups. Overall, there is no significant effect on cardiovascular parameters among the three groups and small, negligible effect sizes were noticed in this study. The ηp^2^ values for the non-significant Group × Time interactions (0.037 for heart rate, 0.013 for systolic blood pressure, 0.008 for diastolic blood pressure, 0.005 for SpO_2_%) are indeed negligible. However, the main effect of time was large (0.626 for heart rate, 0.507 for systolic blood pressure), which is expected due to the exercise.

## Discussion

This study aimed to explore the cardiovascular effects of wearing different Personal Protective Equipment (PPE) while exercising, as examined by a standardized 6-Minute Walk Test (6-MWT), which can assess the functional capacity under regular conditions. Since the beginning of the COVID-19 pandemic, speculation regarding the effects of facemasks or PPE on physiological parameters has been widespread. Many studies have found that physical activity can result in changes in the cardiopulmonary parameters to meet the metabolic demands during exercise.^18^

Previous studies found that reduced cardiopulmonary exercise capacity in participants who prolonged the use of facemasks, including KN95, but during work or varying physical exercise, had not much impact on performance and on physiological variables among subjects of different age and health status.^19^ Studies also found significant differences in heart rate and increased perceived breathing difficulty with N95 facemasks compared to surgical masks, but no significant differences in oxygen saturation were observed between controls and respirator-using groups.^7^ Increased respiratory resistance on wearing N95 masks decreases pulmonary parameters, but myocardial compensation with increased heart rate meets the demand of maximum energy consumption and maximum oxygen uptake (VO_2max_).^7^ In contrast, other studies showed minimal or no variation in heart rate at rest, but an increase in heart rate and a drop of SpO_2_% was observed after physical exertion upon the use of N95.^1^^,^^5^ Moreover, a significant increase in heart rate and blood pressure, and a decrease in SpO_2_% occur only after prolonged use of N95 facemask due to hypoxia and hypercarbia.^3^ The present study showed similar findings within the groups between pretest and posttest, but SpO_2_% did not show a significant difference.

The extent of changes in cardiovascular parameters depends on the intensity of the activity performed and the type of environment in which the activity is executed.^20^^,^^21^ There were no significant changes in SpO_2_% with graded exercise between those with an N95 facemask and without a facemask.^22^ Wearing an N95 facemask for prolonged duration had increased heart rate and decreased SpO_2_%; whereas, those with any type of facemasks performing progressive cycle exercise did not show significant cardiopulmonary changes.^23^^,^^24^

In the COVID-19 era, some researchers suggested using 6-MWT for the discrimination of disease severity and for the early diagnosis of mild cases.^16^ During COVID-19, PPE with no-valve masks caused breathlessness among healthcare workers, but no significant physiological changes when N95 masks with no-valve were used.^25^ However, another study found that, irrespective of the types of masks such as cloth mask, surgical mask, and N95 mask, there were no significant differences in heart rate, SpO_2_%, and blood pressure, except for the feeling of breathing effort while using a cloth mask during the 6-MWT.^26^ Similarly, those with FFP2/N95 had no other cardiopulmonary changes after 6-MWT.^27^ In the present study, after considering the Group x Time interaction, there were no statistically significant differences in cardiovascular parameters before and after 6-MWT among control, facemask and full-body PPE groups. No significant effect of time on physiological parameters among the three groups, small and negligible effect sizes were noticed in this study.

The physical characteristics of the participants, such as their BMI, may influence the outcome of the intervention (6-MWT) and wearing facemask. Studies reported that walking distances either decreased or remained constant with increasing BMI.^28^, ^29^, ^30^, ^31^ In the present study, most of the participants were normal with a mean BMI of 22.79±4.71kg/m^2^ and BMI had not much influence on performing 6-MWT. Participants wearing facemasks walked approximately 30 m farther than those wearing full-body PPE, which might have influenced the physical limitation. In this study 6-Minute Walk Distance (6-MWD) was statistically significantly different between the facemask group and the full-body PPE group, but not with the control group.

Very few studies have examined the influence of full-body PPE on oxygen saturation while performing regular jobs among the general population. In the presenet study, those who wore full-body PPE or facemask did not show a statistically significant difference before and after the test (6-MWT), which is consistent with other study findings of performing 6-MWT, wearing different types of facemasks.^13^^,^^14^^,^^26^^,^^27^ This confirms the authors’ hypothesis that the use of facemasks or full-body PPE would have no statistically significant differences on cardiovascular parameters. The present study investigated the cardiovascular effects of wearing different PPE among young healthy medical students with a brief intervention of submaximal physical activity (6-MWT). The results showed a small effect size. This study adds to the literature by showing that even full-body PPE, not just masks, does not alter the acute cardiovascular response to submaximal exercise in healthy young adults. The scope of this study cohort indicated that any changes in the mean values of each parameter among the groups were minimal, and the results cannot be generalized to prolonged PPE use, strenuous activity, older individuals, or those with comorbidities, populations for whom the clinical question is most demanding.

### Limitation

The homogeneity of the sample was not attained by this quasi-experimental study design as participants were randomly selected and group allocation was concealed prior to the study. Though this study design has susceptibility to measurement bias by assessors who were medical students, bias was limited due to appropriate calibration of equipment, and proper training of assessors to use the equipment during the pilot study. The potential confounding factors such as individual baseline physiological measurements and cardiopulmonary or health fitness, including any recent covid infection or vaccination, were taken into consideration in the inclusion criteria. Though the study was conducted in the same environment for all the groups at one time measurement, daily variations of temperature and humidity might influence physiological responses, especially when wearing full-body PPE. However, the data collected was appropriate for the environment chosen, for the method of physical exertion (6-MWT), and participant characteristics (young, healthy medical students) involved a homogenous population as a major limitation to generalizability.

## Conclusion

This study employed the 6-Minute Walk Test (6-MWT), a standardized method, as an intervention to evaluate the cardiovascular parameters among groups wearing different PPE. The pretest and posttest cardiovascular parameters, such as heart rate, blood pressure, SpO_2_%, and 6-Minute Walk Distance (6-MWD), were compared and analyzed for Group × Time interaction between the groups for any significant differences. In conclusion, the authors found that within the limits of this study's power and design, no statistically significant differences were detected in cardiovascular parameters between the groups wearing full-body PPE or a facemask compared with the control group after a 6-minute walk. This study involved only healthy young adults and tested a single, short-duration submaximal physical activity, a brief 6-minute walk. The results cannot be generalized to prolonged PPE use, strenuous activity, older individuals, or those with comorbidities. Further recommend extending the study involving various populations and different environments for the use of PPE towards the benefit of society.

## Data availability

The datasets analyzed in this study are available from the corresponding author upon request.

## Ethical approval

Ethical approval was obtained from the University Research Ethics Committee, the reference number UNIKL REC/2022/006 for the duration of the study from 1st April 2022 to 31st March 2024.

## Authors' contributions

All authors contributed to the conceptualization of a research topic and the design of the study. Padmavathy, Noorzaid and Resni were involved throughout the study for data collection. Padmavathy and Rohith analyzed after cleaning the entered data, compiled the results and contributed to manuscript drafting. Thengi contributed to the reanalysis and interpretation of data. All authors critically reviewed the manuscript and agreed on the final version to be submitted.

## Funding

This research was supported by a University Short-Term Research Grant of the University of Kuala Lumpur, Royal College of Medicine Perak. (STRG Ref: UniKL/CoRI/str21033, 2022).

## Declaration of competing interest

The authors declare no conflicts of interest.
